# Magnesium Homeostasis in Myogenic Differentiation—A Focus on the Regulation of TRPM7, MagT1 and SLC41A1 Transporters

**DOI:** 10.3390/ijms23031658

**Published:** 2022-01-31

**Authors:** Monica Zocchi, Laura Locatelli, Gian Vincenzo Zuccotti, André Mazur, Daniel Béchet, Jeanette A. Maier, Sara Castiglioni

**Affiliations:** 1 Department of Biomedical and Clinical Sciences Luigi Sacco, Università di Milano, 20157 Milano, Italy; monica.zocchi@unimi.it (M.Z.); laura.locatelli@unimi.it (L.L.); gianvincenzo.zuccotti@unimi.it (G.V.Z.); jeanette.maier@unimi.it (J.A.M.); 2 Pediatric Department, “Vittore Buzzi” Children’s Hospital, 20154 Milano, Italy; 3 UNH—Unité de Nutrition Humaine, Université Clermont Auvergne, INRAE, F-63000 Clermont-Ferrand, France; andre.mazur@inrae.fr (A.M.); daniel.bechet@inrae.fr (D.B.); 4 Interdisciplinary Centre for Nanostructured Materials and Interfaces (CIMaINa), Università di Milano, 20133 Milano, Italy

**Keywords:** myogenesis, C2C12 cells, magnesium, TRPM7, MagT1, SLC41A1, autophagy

## Abstract

Magnesium (Mg) is essential for skeletal muscle health, but little is known about the modulation of Mg and its transporters in myogenic differentiation. Here, we show in C2C12 murine myoblasts that Mg concentration fluctuates during their differentiation to myotubes, declining early in the process and reverting to basal levels once the cells are differentiated. The level of the Mg transporter MagT1 decreases at early time points and is restored at the end of the process, suggesting a possible role in the regulation of intracellular Mg concentration. In contrast, TRPM7 is rapidly downregulated and remains undetectable in myotubes. The reduced amounts of TRPM7 and MagT1 are due to autophagy, one of the proteolytic systems activated during myogenesis and essential for the membrane fusion process. Moreover, we investigated the levels of SLC41A1, which increase once cells are differentiated, mainly through transcriptional regulation. In conclusion, myogenesis is associated with alterations of Mg homeostasis finely tuned through the modulation of MagT1, TRPM7 and SLC41A1.

## 1. Introduction

Magnesium (Mg), the second most abundant cation within the intracellular compartment, plays a crucial role in skeletal muscle cells. Acting as a calcium (Ca) antagonist on Ca-permeable channels, Mg is fundamental in regulating muscle contraction. Indeed, hypomagnesemic patients can develop muscle twitches, tremors and cramps [1].

Skeletal muscle health is strictly related to the regenerative capacity of satellite cells, the myogenic quiescent progenitors responsible for the differentiation of new fibers [2,3,4]. To maintain muscle homeostasis or in response to an injury, satellite cells can be activated to proliferate becoming myoblasts. With the activation of the myogenic program, myoblasts withdraw from the cell cycle, migrate and elongate in order to align with other myoblasts. The elongated myoblasts undergo a rearrangement of actin cytoskeleton at contact sites and a subsequent membrane fusion which induces the formation of multinucleated myotubes.

It has been proposed that Mg might play a role in the myogenic process since Mg deficiency enhances the expression of the myogenic proteins Myogenic Differentiation 1 (MyoD) and Myogenin (Myog) in rat skeletal muscle [5]. Moreover, we have recently demonstrated that non-physiological low or high extracellular Mg concentrations inhibit myogenesis in C2C12 myoblasts, through an ROS-mediated impairment of membrane fusion [6].

At the cellular level, Mg homeostasis is finely regulated by the action of transmembrane channels and transporters, which in the last years have arisen a lot of attention for their involvement in several pathophysiological processes. Some of them are ubiquitously expressed in almost all the tissues, such as Transient Receptor Potential Cation Channel Subfamily M Member 7 (TRPM7) [7,8], Magnesium Transporter 1 (MagT1) [9] and Solute Carrier family 41 member A1 (SLC41A1) [10].

While TRPM7 is characterized by an ion channel mainly permeable to Ca and Mg cations and an α-kinase domain, MagT1 is highly specialized for Mg transport across the plasma membrane. SLC41A1 contributes to the maintenance of cellular Mg homeostasis through complex mechanisms not completely unveiled [11].

Few data are available concerning Mg transporters in skeletal muscle. TRPM7 has been shown to be involved in the regulation of vascular smooth muscle cells function [12] as well as in modulating action potential of cardiomyocytes [13]. Nonetheless, the function of TRPM7 in skeletal muscle physiology and muscle cell differentiation has not been elucidated yet. SLC41A1 is expressed in smooth muscle cells and has a role in regulating their proliferation and migration [14], while presently no data are available for MagT1. As for TRPM7, it is still obscure whether SLC41A1 and MagT1 play a role in myogenic differentiation and in mature skeletal muscle.

To fulfil this knowledge gap, here we analyzed the physiological modulation of intracellular Mg during myogenesis and the expression of TRPM7, SLC41A1 and MagT1 in C2C12, an in vitro model of murine myoblasts which are able to efficiently differentiate in myotubes under specific culture conditions [15]. Our aim was to understand whether these Mg transporters could be actively involved in myogenic differentiation, thus discovering new potential functions in skeletal muscle physiology.

## 2. Results

### 2.1. Mg Concentration Decreases during Myogenesis and Is Restored in Myotubes

Although some studies demonstrated the importance of Mg in skeletal muscle maintenance, little remains known about Mg homeostasis during skeletal muscle formation. On this basis, we analyzed the intracellular Mg concentration during myogenesis.

**Figure 1:**
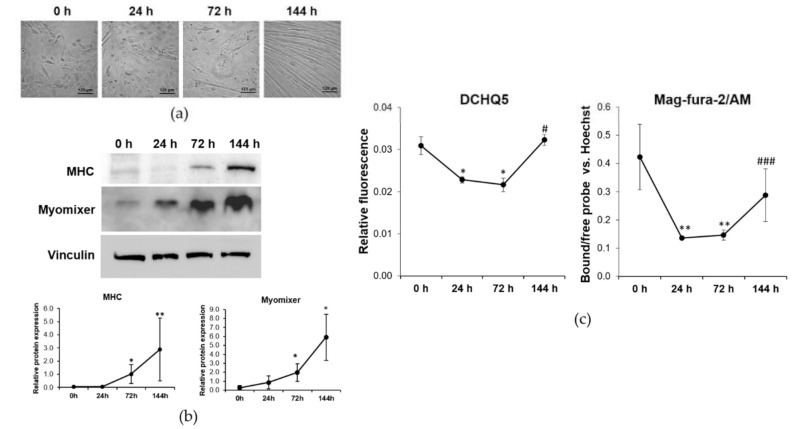
Intracellular Mg concentration is modulated during myogenesis. C2C12 were induced to differentiate for 144 h in DM. (**a**) Pictures were taken with optical microscope (10× magnification) at different time points. (**b**) MHC and Myomixer expression was analyzed by western blot. Vinculin was used as control of loading. A representative blot (upper panel) and densitometry performed on three independent experiments and obtained by ImageLab (low panel) are shown. (**c**) Total Mg was measured using the fluorescent chemosensor DCHQ5. Free Mg was measured using Mag-fura-2/AM. Non-parametric Mann–Whitney test was performed to define statistical significance among the time points. * Indicates significance compared to 0 h. # Indicates significance compared to the previous time point. */# *p* ≤ 0.05; ** *p* ≤ 0.01; ### *p* ≤ 0.001.

C2C12 were induced to differentiate using a differentiation medium (DM) containing 2% horse serum, a process that requires 144 h under our experimental conditions [6]. Cell differentiation was monitored by optical microscopy. As shown in Figure 1a, the first elongated cells appear after 72 h of culture in DM, while after 144 h a large number of thick multinucleated myotubes are detected on all the well surface.

By western blot, we analyzed the expression of Myomixer, a key peptide involved in fusion pores formation [16], and found it significantly upregulated starting from 72 h of culture in DM (Figure 1b). The contractile protein Myosin Heavy Chain (MHC), which is only expressed in differentiated myotubes, follows a similar kinetic, being increased at 72 h and, much more, at 144 h of culture in DM.

At the different time points of differentiation, we measured the intracellular concentrations of Mg. As shown in Figure 1c, the concentrations of both total and free Mg decrease after 24 and 72 h while after 144 h from induction of the differentiation process the concentrations of Mg return to the initial levels measured in C2C12 before myogenesis induction (0 h).

### 2.2. Mg Transporters Are Differently Modulated during Myogenesis

Mg homeostasis is maintained inside the cells by a number of specialized membrane transporters, such as MagT1 and SLC41A1, and ion channels among which TRPM7. We asked whether these transporters might have a role in the modulation of Mg concentration during the myogenic differentiation of C2C12. Therefore, we analyzed the expression of these Mg transporters/channels both at mRNA and protein levels in C2C12 induced to differentiate.

**Figure 2:**
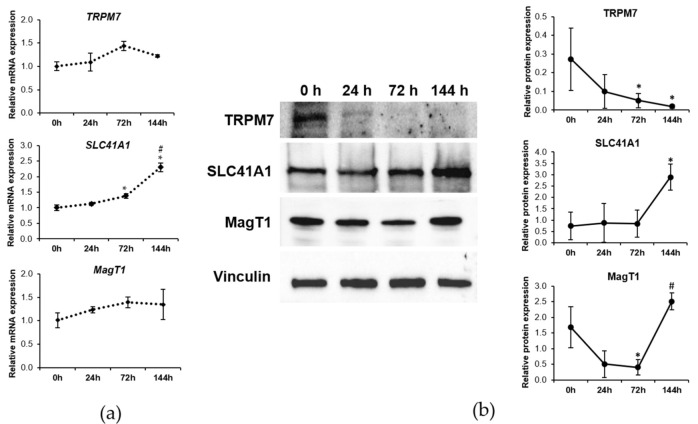
Mg transporters are differently expressed during myogenesis. C2C12 were induced to differentiate up to 144 h. The mRNA expression and protein levels of TRPM7, SLC41A1 and MagT1 were analyzed by Real-time PCR (**a**) and western blot (**b**), respectively. Vinculin was used as control of loading. A representative blot and densitometry performed on three independent experiments and obtained by ImageLab are shown in (**b**). Non-parametric Mann–Whitney test was performed to define statistical significance among the time points. * Indicates significance compared to 0 h. # Indicates significance compared to the previous time point. */# *p* ≤ 0.05.

Although we did not find any significant modulation of TRPM7 at mRNA level (Figure 2a), by western blot we demonstrated a gradual decrease of the protein content which started after 24 h from myogenesis induction (Figure 2b). In multinucleated myotubes, no TRPM7 is detected.

SLC41A1 expression increases both at mRNA and protein levels after 144 h of exposure to DM, when myotubes are formed (Figure 2a,b).

Regarding MagT1, no significant modulation at mRNA level was detected, whereas the protein levels were reduced starting 24 h after myogenic induction to become significant after 72 h. When myotubes are formed after 144 h, MagT1 returns to the initial expression level (Figure 2a,b).

### 2.3. Proteasome, Calpains, and Autophagy Are Activated during Myogenesis

On the basis of aforementioned data, it is reasonable to assume that both TRPM7 and MagT1 undergo post-translational regulation during myogenesis.

Some proteolytic pathways are important for both muscle wasting and regeneration, among which include calpains, the ubiquitin-proteasome and the autophagy-lysosome systems.

**Figure 3:**
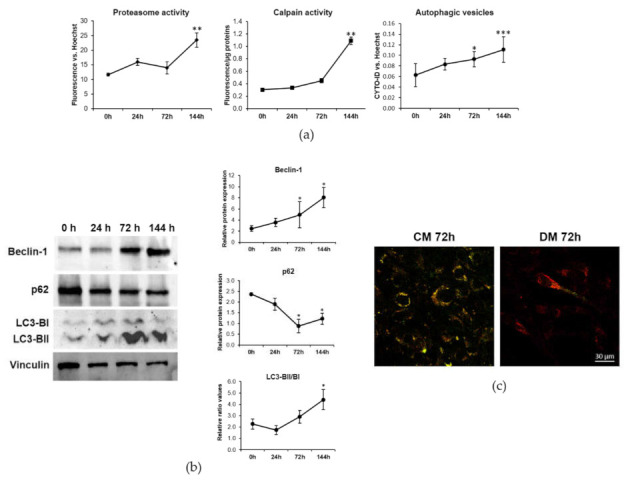
Calpains, proteasome and autophagy are activated during myogenesis. C2C12 were induced to differentiate in DM. (**a**) Proteasome and Calpain activity and autophagic vesicles were measured as described in the methods. (**b**) The amounts of Beclin-1, p62 and LC3-BII/LC3-BI were analyzed by western blot. Vinculin was used as control of loading. A representative blot (left panel) and densitometry performed on three independent experiments and obtained by ImageLab (right panel) are shown. (**c**) Autophagic flux imaging was performed by the Tandem fluorescent-tagged LC3 assay as described in the methods. Non-parametric Mann–Whitney test was performed to define statistical significance among the time points. * Indicates significance compared to 0 h. * *p* ≤ 0.05; ** *p* ≤ 0.01; *** *p* ≤ 0.001.

Initially, we analyzed the activity of these three pathways during the myogenic differentiation of C2C12. Figure 3a shows a significant increase of proteasome activity at 144 h. Calpain activity progressively increases during myogenesis, reaching significantly higher levels after 144 h of cell differentiation. Concerning autophagy, we detected a significant increase in the amount of autophagic vesicles after 72 and 144 h from the induction of myogenesis (Figure 3a).

We additionally analyzed by western blot the expression of some autophagic proteins during the progression of myogenesis (Figure 3b). After 72 and 144 h we found the marked induction of Beclin-1, which contributes to the initiation of autophagosomes formation by interacting with phosphatidylinositol 3-kinase. Moreover, at these time points we detected the conversion of microtubule-associated protein 1A/1B light-chain phosphatidylethanolamine conjugate (LC3-BI) to autophagosome-associated LC3-BII, which is the most widely used autophagosome marker. The cargo adaptor protein p62 is responsible for delivering the autophagic substrates to autophagosomes, being itself degraded in the process. The downmodulation of p62 expression confirms the activation of the autophagic process during C2C12 differentiation.

To reinforce these results, we performed a Tandem fluorescent-tagged LC3 assay to monitor the autophagic flow. The quenching of Green Fluorescent Protein (GFP) fluorescence and the increase of Red Fluorescent Protein (RFP) one in C2C12 after 72 h of culture in DM suggests an increase in the fusion of autophagosomes with lysosomes, highlighting that the autophagic process is functional and highly active in myogenesis (Figure 3c).

### 2.4. Autophagy Is Responsible for the Downregulation of TRPM7 and MagT1

In spite of no modulation of their transcripts, TRPM7 and MagT1 are significantly downregulated at the protein level after 72 h of myogenic induction. Therefore, we explored the involvement of post-translational events in regulating their content.

We cultured C2C12 for 72 h in their standard culture medium (CM) or in DM in the presence of the proteasome inhibitor MG132, the calpain inhibitor calpeptin or the autophagic flux inhibitor bafilomycin and we assessed the Mg transporters’ levels by western blot.

**Figure 4:**
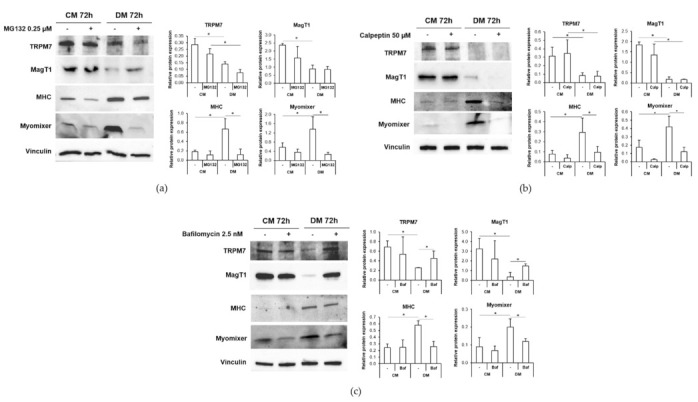
Autophagy inhibition restores TRPM7 and MagT1 expression during myogenesis. C2C12 were cultured in CM or DM for 72 h in the presence of the proteasome inhibitor MG132 (0.25 µM) (**a**) or calpeptin (50 µM) (**b**) or bafilomycin (2.5 nM) (**c**). TRPM7, MagT1, MHC and Myomixer expression was analyzed by western blot. Vinculin was used as control of loading. A representative blot (left panel) and densitometry performed on three independent experiments and obtained by ImageLab (right panel) are shown. To define statistical significance, non-parametric Kruskal–Wallis test was used and the p-values were corrected using the Dunn’s method. * *p* ≤ 0.05.

As shown in Figure 4, the three inhibitors do not modulate the expression of TRPM7 and MagT1 in control cells cultured in CM. After 72 h of culture in DM, only the treatment with bafilomycin (Figure 4c) prevents the downmodulation of both TRPM7 and MagT1, indicating that the two Mg transporters undergo proteolysis during myogenesis through autophagy. Neither the inhibition of proteasome (Figure 4a) nor the suppression of calpain activity (Figure 4b) rescue their content.

Moreover, MG132, calpeptin or bafilomycin inhibited myogenesis, as shown by the reduced expression of MHC in the cells cultured in DM in the presence of the inhibitors. Interestingly, all three inhibitors prevent the upregulation of Myomixer and, hence, membrane fusion.

## 3. Discussion

A correct Mg homeostasis is fundamental for skeletal muscle health. Given the role of Mg as a Ca antagonist, a low Mg status is correlated with skeletal muscle hyper contractibility [1,17]. Moreover, muscles exhibit an altered expression of Mg transporters in older men [18] and the low-grade inflammatory state connected to hypomagnesemia may contribute to the development of sarcopenia [19,20,21]. For these reasons, Mg transport and homeostasis regulation in skeletal muscle need to be investigated in further detail. We have recently demonstrated in a murine model that even a short-term mild low Mg diet is able to induce a significant reprogramming of several genetic pathways involved in skeletal muscle physiology, regeneration and metabolism [22]. In the muscle of hypomagnesemic mice, a significant downregulation of Mg transporters has been observed. Moreover, we demonstrated that Mg is required to accomplish myogenic differentiation and membrane fusion since non-physiological extracellular Mg concentrations impact the myoblasts ability to fuse and form multinucleated cells [6]. Nonetheless, very little remains known about Mg homeostasis and its transporters in myogenesis.

Proteins mediating Mg transport have been shown to be implicated in differentiation. We have previously demonstrated that both TRPM7 and MagT1 contribute to osteogenic differentiation of human bone marrow-derived mesenchymal stem cells (hMSC) [23,24]. MagT1 is additionally involved in odontogenic differentiation of hMSC [25]. TRPM7 has been shown to play a pivotal role on differentiation of SH-SY5Y neuronal cell line [26] and human erythromyeloid leukemia cell line K562 [27], and its inhibition prevents differentiation of human lung fibroblasts [28]. These data demonstrate the importance of studying the role of these transporters in differentiation.

In our model of myogenesis, the first elongated cells appear after 72 h of culture in DM and after 144 h a large number of thick and contractile myotubes are visible by optical microscopy, a result confirmed by the upregulation of MHC expression. While differentiating, a significant decrease of total and free intracellular Mg was observed in C2C12 cells, whereas once myotubes are formed the intracellular concentration of Mg raises and reverts to basal levels. We therefore argue that modulation of the amounts of proteins involved in Mg homeostasis might explain these results. We focused on three well described regulators of intracellular Mg levels, i.e., TRPM7, MagT1 and SCL41A1 [1].

After 72 h of differentiation, a significant downregulation of TRPM7 and MagT1 is observed. While MagT1 initially decreases to return to basal levels in myotubes, TRPM7 remains downregulated. These results suggest that MagT1 is fundamental in restoring intracellular Mg concentrations and point to its fundamental role in Mg homeostasis in C2C12 cells. The downregulation of TRPM7 is in agreement with results showing that in human skeletal muscle TRPM7 protein is undetectable (https://www.proteinatlas.org/ENSG00000092439-TRPM7/tissue (accessed on 20 December 2021)), while MagT1 and SLC41A1 are appreciable. It might be concluded that TRPM7 downregulation is a marker of muscle differentiation.

SLC41A1 expression is not modulated in the early phases of myogenesis and is upregulated in myotubes. We hypothesize that the increased amounts of SLC41A1 might finely tune intracellular Mg concentrations in myotubes.

By the analysis of mRNA and protein levels, we propose that SLC41A1 is transcriptionally regulated, while TRPM7 and MagT1 undergo post-translational regulation during myogenesis. These results underscore that it is imperative to evaluate the modulation of proteins, since, even if no alterations of the levels of the transcripts occur, the amounts of the corresponding proteins can change due to post-transcriptional or post-translational events.

It is interesting to underline that TRPM7 and MagT1 are degraded after 72 h from myogenesis induction when membrane fusion starts to occur, as verified by the upregulation of the newly discovered fusogenic peptide Myomixer [16]. This suggests that Mg transport systems undergo a significant rearrangement during membrane fusion and multinucleated myotubes formation. In particular, to understand the mechanisms involved in TRPM7 and MagT1 downmodulation, we investigated the proteolytic pathways activated during myogenesis [29]. In agreement with previous data, we confirm in our model that calpains, proteasome and autophagy-lysosome systems are activated during the C2C12 differentiation process.

Calpains seem to be implicated in some aspects of myogenesis. In particular, some studies demonstrated that calpain inhibition affects the myoblast fusion of C2C12 [30] and induces the formation of thinner myotubes containing a reduced number of nuclei in chick myogenic cells [31]. Moreover, Calpains are involved in the downmodulation of protein kinase C (PKC) during myoblast differentiation. PKC is responsible for the phosphorylation of the myogenic factor Myog which, upon phosphorylation, loses the ability to activate the muscle specific genes resulting in the repression of myogenesis [32]. Coherently, we detected a significant downregulation of MHC and Myomixer levels in C2C12 induced to differentiate in the presence of calpeptin, confirming the fundamental role of calpains in the fusion process during myogenesis. However, calpain inhibition did not prevent the downregulation of TRPM7 and MagT1 that occurs in the cells cultured in DM, thus suggesting the involvement of a different mechanism in Mg transporters modulation in C2C12.

It is known that the ubiquitin-proteasome pathway plays an essential role in myogenesis especially in the transcriptional control of myogenic and cell cycle regulators and in the control of myoblast fusion. The proteasome inhibitor lactacystin causes the activation of p21, which is responsible for cell cycle withdrawal, and the expression of MyoD, Myogenin and Retinoblastoma (Rb) without occurring the myoblast fusion. Probably, the induction of p21 prior to MyoD is responsible for myogenic repression in the presence of a proteasome inhibitor [33]. Our data confirm the role of proteasome in myogenesis since its inhibition impairs the expression of both MHC and Myomixer. However, proteasome inhibition is not sufficient to prevent TRPM7 and MagT1 downregulation in differentiating C2C12 cells.

The autophagy-lysosome system has been demonstrated to be essential for muscle physiology [34]. A fine tuning of autophagy is fundamental to allow the correct development of myotubes as it guarantees a time-related connection between the synthesis of differentiation-associated proteins and the fusion process [35]. Indeed, autophagy is induced during myogenesis and its inhibition impairs the differentiation and fusion of C2C12 and favors their apoptosis [35]. Here we additionally demonstrate a concomitance between autophagy induction and the fusogenic peptide Myomixer upregulation during myogenesis. Indeed, autophagy inhibition downregulates Myomixer expression which might result in fusion impairment. Importantly, here we demonstrate for the first time that autophagy is directly responsible for the degradation of the Mg transporters TRPM7 and MagT1 during myogenesis, since the autophagy inhibitor bafilomycin restores the levels of the two transporters in DM.

These data offer new insights into the mechanisms involved in the complex regulation of Mg homeostasis in skeletal muscle biogenesis.

## 4. Materials and Methods

### 4.1. Cell Culture

C2C12 murine myoblasts were purchased from Sigma-Aldrich (St. Louis, MO, USA). Once they reached 50% of confluence, the cells were serially passaged in culture medium (CM) composed of DMEM high glucose added with 20% of heat-inactivated fetal bovine serum (FBS), glutamine (2 mM) and 1% penicillin/streptomycin (Euroclone S.p.A., Pero, Italy).

To induce myogenic differentiation, confluent myoblasts were cultured in differentiation medium (DM) consisting of DMEM high glucose added with 2% horse serum.

To inhibit proteolytic systems during differentiation, cells were treated in CM and DM for 72 h with the following compounds: MG132 (Sigma-Aldrich, St. Louis, MO, USA) was used at 0.25 µM to inhibit proteasome activity; calpeptin (Sigma-Aldrich, St. Louis, MO, USA) was used at 50 µM to inhibit calpain activity; bafilomycin (Sigma-Aldrich, St. Louis, MO, USA) was used at 2.5 nM to inhibit autophagic flux.

Images of cultured cells were acquired by optical microscopy with FLoid™ Cell Imaging Station (Thermo Fisher Scientific, Waltham, MA, USA) with 10× magnification.

### 4.2. Mg Concentrations Measurement

DCHQ5 is a Mg-selective fluorescent chemosensor which detects and quantifies intracellular total Mg, measuring both free cation and macromolecules-bound Mg pools [36]. After 0, 24, 72 and 144 h of culture in DM, C2C12 were collected, counted and 10,000 cells were used for the analysis. Cells were lysed in Phosphate Buffer Saline (PBS) and sonicated. Then the sample was diluted in a 1:1 MOPS (3-(*N*-morpholino) propanesulfonic acid): MeOH (pH 7.4) solution and DCHQ5 probe was added at a final concentration of 15 µM. The fluorescent signal (λex = 360 nm, λem = 510 nm) was detected with a Varioskan LUX Multimode Microplate Reader (Thermo Fisher Scientific, Waltham, MA, USA). The experiment was performed three times in triplicate.

Free intracellular Mg was measured with the fluorescent probe Mag-fura-2/AM (Molecular probes, Thermo Fisher Scientific, Waltham, MA, USA). Cells were seeded on a 96-wells black plate (Costar, Sigma-Aldrich, St. Louis, MO, USA) and induced to differentiate. After 0, 24, 72 and 144 h in DM, the cells were incubated for 1 h with the probe at the final concentration of 2.5 µM and the fluorescent signal of the bound and not-bound probe was acquired (bound: λex = 335 nm, λem = 510 nm; not-bound: λex = 370 nm, λem = 510 nm). Each bound-related value was normalized on the respective not-bound value and on total nuclei fluorescent staining with Hoechst 33342 (Thermo Fisher Scientific, Waltham, MA, USA). The experiment was performed three times in triplicate.

### 4.3. Real-Time PCR

Total mRNA was extracted with the PureLink RNA Mini kit (Thermo Fisher Scientific, Waltham, MA, USA). Single-stranded cDNA was synthesized from 0.5–1 µg mRNA in a final volume of 20 µL using High-Capacity cDNA Reverse Transcription Kit (Thermo Fisher Scientific, Waltham, MA, USA) according to manufacturer’s instructions. Real-time PCR was performed on 20 ng of cDNA using TaqMan™ Fast Universal PCR Master Mix (Thermo Fisher Scientific, Waltham, MA, USA) and TaqMan Gene Expression Assays (FAM) (Thermo Fisher Scientific, Waltham, MA, USA). The following primers were used: *TRPM7* (Mm00457998), *SLC41A1* (Mm00715604_m1), and *MAGT1* (Mm00482432_m1). The housekeeping gene *GAPDH* (Mm99999915_g1) was used as an internal reference gene. The reactions were performed and analyzed with CFX96 Real-Time PCR Detection System (Bio-Rad, Hercules, CA, USA). Relative changes in gene expression were analyzed with the 2^−ΔΔCt^ method, considering 0 h (= 1) values as reference.

### 4.4. SDS-PAGE and Western Blot

Total protein extracts were obtained with a lysis buffer (50 mM Tris-HCl pH 7.4, 150 mM NaCl, 1% NP-40, 0.25% Na-deoxycholate) added with protease inhibitors. Total protein extracts were quantified with Bradford assay and 20–40 µg of proteins were separated by SDS-PAGE on Mini-PROTEAN TGX Stain-free Gels (Bio-Rad, Hercules, CA, USA) and transferred to nitrocellulose membranes by using Trans-Blot^®^ TurboTM Transfer Pack (Bio-Rad, Hercules, CA, USA). After blocking with a solution of 5% bovine serum albumin (BSA) (Sigma-Aldrich, St. Louis, MO, USA), western blot analysis was performed using primary antibodies against Myosin Heavy Chain (MHC) (1:1000), Myomixer (ESGP) (1:2000) (R&D Systems, MN, USA), TRPM7 (1:1000) (Bethyl, Montgomery, TX, USA), SLC41A1 (1:1000) (Thermo Fisher Scientific, Waltham, MA, USA), MagT1 (1:1000) (Abcam, Cambridge, UK), LC3B (1:1000), Beclin-1 (1:1000) (Cell Signaling, Danvers, MA, USA), p62 (1:1000) (Thermo Fisher Scientific, Waltham, MA, USA) and Vinculin (1:3000) (Sigma-Aldrich, St. Louis, MO, USA). After three washes of 10 min each in Tris Buffer Saline added with 1% Tween (TBS-T), the membranes were incubated with secondary antibodies conjugated with horseradish peroxidase (Amersham Pharmacia Biotech Italia, Cologno Monzese, Italy). After three washes of 10 min each in TBS-T, the immunoreactive proteins were detected with ClarityTM Western ECL substrate (Bio-Rad, Hercules, CA, USA) and images were acquired with the ChemiDoc MP Imaging System (Bio-Rad, Hercules, CA, USA). Time of exposure was determined automatically by the ChemiDoc and set to prevent signal saturation.

Densitometry of the bands was performed with the software ImageLab (Bio-Rad, Hercules, CA, USA). Vinculin was considered a control of loading and its expression was used to normalize the densitometric quantification of all the proteins analyzed. In the figures, representative blots are shown, while the densitometric analysis was performed at least on three independent experiments.

### 4.5. Proteasome, Calpain and Autophagic Flux Analysis

The fluorometric assays were conducted according to manufacturers’ instructions and fluorescence was acquired at Varioskan LUX Multimode Microplate Reader (Thermo Fisher Scientific, Waltham, MA, USA).

To study proteasome activity, cells were seeded on a 96-wells black plate (Costar, Sigma-Aldrich, St. Louis, MO, USA) and induced to differentiated in DM. After 0, 24, 72 and 144 h, 20S Proteasome Activity Assay (Sigma-Aldrich, St. Louis, MO, USA) was used. Fluorescence was acquired at λex = 490 nm, λem = 525nm. Nuclei staining with Hoechst 33342 (Thermo Fisher Scientific, Waltham, MA, USA) (λex = 361nm, λem = 497 nm) was performed for signal normalization.

To measure calpain activity, cells were seeded on a 6-wells plate (Costar, Sigma-Aldrich, St. Louis, MO, USA) and collected after 0, 24, 72 and 144 h of differentiation in DM to be analyzed using the Calpain Activity Assay Kit (Sigma-Aldrich, St. Louis, MO, USA). Cell lysates were obtained with Extraction Buffer and protein quantification was performed with the Bradford reagent (Sigma-Aldrich, St. Louis, MO, USA). Each reaction was set up starting from 30–50 µg of lysates. The fluorescence (λex = 400 nm, λem = 505 nm) was normalized on protein amount.

To study autophagy, CYTO-ID autophagy detection kit (Enzo Life Sciences, Euroclone S.p.A., Pero, Italy) was used. The cells were seeded on a 96-wells black plate (Costar, Sigma-Aldrich, St. Louis, MO, USA) and after 0, 24, 72 and 144 h in DM they were incubated with CYTO-ID detection reagent (λex = 480 nm, λem = 530 nm) for autophagic vesicles staining and co-stained with Hoechst 33342 (Thermo Fisher Scientific, Waltham, MA, USA) for nuclear detection and fluorescence normalization.

Each assay was performed at least in triplicate and repeated three times.

### 4.6. Autophagy Tandem Sensor and Confocal Imaging

Cells were seeded on glass coverslips and cultured in CM and DM for 72 h. The day after seeding, the cells were transfected with Premo™ Autophagy Tandem Sensor RFP-GFP-LC3B Kit (Molecular Probes, Thermo Fisher Scientific, Waltham, MA, USA) according to manufacturer’s instructions, and fixed in PBS containing 4% paraformaldehyde and 2% sucrose, pH 7.6. The principle of this assay resides in the fact that the low pH in the lysosomes quenches the fluorescence of GFP, whereas the fluorescence of RFP remains stable. Upon the formation of autophagosomes the number of GFP-positive/RFP-positive (yellow) vesicles is increased. These vesicles become GFP-negative/RFP-positive (red) after fusion with lysosomes.

Finally, the coverslips were mounted using ProLongTM Gold antifade reagent (Thermo Fisher Scientific, Waltham, MA, USA). Images were acquired with a SP8 Leica confocal microscope using a 40× objective in oil.

### 4.7. Statistical Analysis

Data are expressed as the mean ± standard deviation. Data were non-parametric according to D’Agostino–Pearson normality test. For single comparisons, statistical significance was evaluated with non-parametric unpaired Mann–Whitney test. For multiple comparisons, Kruskal–Wallis test was used and the p-values were corrected using the Dunn’s method. The statistical analysis was performed with the software GraphPad Prism. In the figures, * indicates significance compared to 0 h and # compared to the previous time point. Statistical significance was defined as *p*-value ≤ 0.05 and in particular: */# *p* ≤ 0.05; **/## *p* ≤ 0.01; ***/### *p* ≤ 0.001.

## Data Availability

The data presented in this study are openly available in Dataverse at https://dataverse.unimi.it/dataverse/IJMS-Muscle (accessed on 28 December 2021).

## References

[1] de Baaij J.H.F., Hoenderop J.G.J., Bindels R.J.M. (2015). Magnesium in Man: Implications for Health and Disease. Physiol. Rev..

[2] Yusuf F., Brand-Saberi B. (2012). Myogenesis and muscle regeneration. Histochem. Cell Biol..

[3] Wang J., Conboy I. (2010). Embryonic vs. adult myogenesis: Challenging the “regeneration recapitulates development” paradigm. J. Mol. Cell Biol..

[4] Le Grand F., Rudnicki M.A. (2007). Skeletal muscle satellite cells and adult myogenesis. Curr. Opin. Cell Biol..

[5] Furutani Y., Funaba M., Matsui T. (2011). Magnesium deficiency up-regulates Myod expression in rat skeletal muscle and C2C12 myogenic cells. Cell Biochem. Funct..

[6] Zocchi M., Béchet D., Mazur A., Maier J.A., Castiglioni S. (2021). Magnesium influences membrane fusion during myogenesis by modulating oxidative stress in c2c12 myoblasts. Nutrients.

[7] Fonfria E., Murdock P.R., Cusdin F.S., Benham C.D., Kelsell R.E., McNulty S. (2006). Tissue distribution profiles of the human TRPM cation channel family. J. Recept. Signal Transduct..

[8] Kunert-Keil C., Bisping F., Krüger J., Brinkmeier H. (2006). Tissue-specific expression of TRP channel genes in the mouse and its variation in three different mouse strains. BMC Genom..

[9] Wolf F.I., Trapani V. (2011). MagT1: A highly specific magnesium channel with important roles beyond cellular magnesium homeostasis. Magnes. Res..

[10] Kolisek M., Launay P., Beck A., Sponder G., Serafini N., Brenkus M., Froschauer E.M., Martens H., Fleig A., Schweigel M. (2008). SLC41A1 is a novel mammalian Mg2+ carrier. J. Biol. Chem..

[11] Arjona F.J., Latta F., Mohammed S.G., Thomassen M., van Wijk E., Bindels R.J.M., Hoenderop J.G.J., de Baaij J.H.F. (2019). SLC41A1 is essential for magnesium homeostasis in vivo. Pflug. Arch..

[12] Touyz R.M., He Y., Montezano A.C.I., Yao G., Chubanov V., Gudermann T., Callera G.E. (2006). Differential regulation of transient receptor potential melastatin 6 and 7 cation channels by ANG II in vascular smooth muscle cells from spontaneously hypertensive rats. Am. J. Physiol. Regul. Integr. Comp. Physiol..

[13] Gwanyanya A., Andriulė I., Istrate B.M., Easmin F., Mubagwa K., Mačianskienė R. (2021). Modulation of the Cardiac Myocyte Action Potential by the Magnesium-Sensitive TRPM6 and TRPM7-like Current. Int. J. Mol. Sci..

[14] Wang D., Zhu Z.-L., Lin D.-C., Zheng S.-Y., Chuang K.-H., Gui L.-X., Yao R.-H., Zhu W.-J., Sham J.S.K., Lin M.-J. (2021). Magnesium Supplementation Attenuates Pulmonary Hypertension via Regulation of Magnesium Transporters. Hypertens.

[15] Burattini S., Ferri R., Battistelli M., Curci R., Luchetti F., Falcieri E. (2004). C2C12 murine myoblasts as a model of skeletal muscle development: Morpho-functional characterization. Eur. J. Histochem..

[16] Bi P., Ramirez-Martinez A., Li H., Cannavino J., McAnally J.R., Shelton J.M., Sánchez-Ortiz E., Bassel-Duby R., Olson E.N. (2017). Control of muscle formation by the fusogenic micropeptide myomixer. Science.

[17] Knochel J.P., Cronin R.E. (1984). The myopathy of experimental magnesium deficiency. Adv. Exp. Med. Biol..

[18] Coudy-Gandilhon C., Gueugneau M., Taillandier D., Combaret L., Polge C., Roche F., Barthélémy J.-C., Féasson L., Maier J.A., Mazur A. (2019). Magnesium transport and homeostasis-related gene expression in skeletal muscle of young and old adults: Analysis of the transcriptomic data from the PROOF cohort Study. Magnes. Res..

[19] Welch A.A., Skinner J., Hickson M. (2017). Dietary magnesium may be protective for aging of bone and skeletal muscle in middle and younger older age men and women: Cross-sectional findings from the UK biobank cohort. Nutrients.

[20] Welch A.A., Kelaiditi E., Jennings A., Steves C.J., Spector T.D., MacGregor A. (2016). Dietary Magnesium Is Positively Associated with Skeletal Muscle Power and Indices of Muscle Mass and May Attenuate the Association between Circulating C-Reactive Protein and Muscle Mass in Women. J. Bone Miner. Res..

[21] Dominguez L.J., Barbagallo M., Lauretani F., Bandinelli S., Bos A., Corsi A.M., Simonsick E.M., Ferrucci L. (2006). Magnesium and muscle performance in older persons: The InCHIANTI study. Am. J. Clin. Nutr..

[22] Bayle D., Coudy-gandilhon C., Gueugneau M., Castiglioni S., Zocchi M., Maj-zurawska M., Palinska-saadi A., Mazur A., Daniel B., Maier J.A. (2021). Magnesium Deficiency Alters Expression of Genes Critical for Muscle Magnesium Homeostasis and Physiology in Mice. Nutrients.

[23] Castiglioni S., Romeo V., Locatelli L., Cazzaniga A., Maier J.A.M. (2018). TRPM7 and MagT1 in the osteogenic differentiation of human mesenchymal stem cells in vitro. Sci. Rep..

[24] Castiglioni S., Romeo V., Locatelli L., Zocchi M., Zecchini S., Maier J.A.M. (2019). The simultaneous downregulation of TRPM7 and MagT1 in human mesenchymal stem cells in vitro: Effects on growth and osteogenic differentiation. Biochem. Biophys. Res. Commun..

[25] Zheng J.-M., Kong Y.-Y., Li Y.-Y., Zhang W. (2019). MagT1 regulated the odontogenic differentiation of BMMSCs induced byTGC-CM via ERK signaling pathway. Stem Cell Res. Ther..

[26] Öz A., Çelik Ö. (2022). The effects of neuronal cell differentiation on TRPM7, TRPM8 and TRPV1 channels in the model of Parkinson’s disease. Neurol. Res..

[27] Takahashi K., Umebayashi C., Numata T., Honda A., Ichikawa J., Hu Y., Yamaura K., Inoue R. (2018). TRPM7-mediated spontaneous Ca(2+) entry regulates the proliferation and differentiation of human leukemia cell line K562. Physiol. Rep..

[28] Yu M., Huang C., Huang Y., Wu X., Li X., Li J. (2013). Inhibition of TRPM7 channels prevents proliferation and differentiation of human lung fibroblasts. Inflamm. Res..

[29] Bell R.A.V., Al-Khalaf M., Megeney L.A. (2016). The beneficial role of proteolysis in skeletal muscle growth and stress adaptation. Skelet. Muscle.

[30] Kumar A., Shafiq S., Wadgaonkar R., Stracher A. (1992). The effect of protease inhibitors, leupeptin and E64d, on differentiation of C2C12 myoblasts in tissue culture. Cell. Mol. Biol..

[31] Buffolo M., Batista Possidonio A.C., Mermelstein C., Araujo H. (2015). A conserved role for calpains during myoblast fusion. Genesis.

[32] Liang Y.-C., Yeh J.-Y., Forsberg N.E., Ou B.-R. (2006). Involvement of mu- and m-calpains and protein kinase C isoforms in L8 myoblast differentiation. Int. J. Biochem. Cell Biol..

[33] Mugita N., Honda Y., Nakamura H., Fujiwara T., Tanaka K., Omura S., Shimbara N., Ogawa M., Saya H., Nakao M. (1999). The involvement of proteasome in myogenic differentiation of murine myocytes and human rhabdomyosarcoma cells. Int. J. Mol. Med..

[34] Bechet D., Tassa A., Taillandier D., Combaret L., Attaix D. (2005). Lysosomal proteolysis in skeletal muscle. Int. J. Biochem. Cell Biol..

[35] Fortini P., Ferretti C., Iorio E., Cagnin M., Garribba L., Pietraforte D., Falchi M., Pascucci B., Baccarini S., Morani F. (2016). The fine tuning of metabolism, autophagy and differentiation during in vitro myogenesis. Cell Death Dis..

[36] Sargenti A., Farruggia G., Zaccheroni N., Marraccini C., Sgarzi M., Cappadone C., Malucelli E., Procopio A., Prodi L., Lombardo M. (2017). Synthesis of a highly Mg(2+)-selective fluorescent probe and its application to quantifying and imaging total intracellular magnesium. Nat. Protoc..

